# Parenting Strategies Used by Parents of Children with ASD: Differential Links with Child Problem Behaviour

**DOI:** 10.1007/s10803-019-04219-2

**Published:** 2019-11-01

**Authors:** Elizabeth O’Nions, Eva Ceulemans, Francesca Happé, Paul Benson, Kris Evers, Ilse Noens

**Affiliations:** 1 Parenting and Special Education Research Unit, Faculty of Psychology and Educational Sciences, KU Leuven, Leuven, Belgium; 2 Leuven Autism Research (LAuRes), KU Leuven, Leuven, Belgium; 3 Developmental Risk & Resilience Unit, Clinical, Educational, and Health Psychology Research Department, Division of Psychology and Language Sciences, University College London, London, UK; 4 Quantitative Psychology and Individual Differences, Faculty of Psychology and Educational Sciences, KU Leuven, Leuven, Belgium; 5 MRC Social, Genetic and Developmental Psychiatry Centre, Institute of Psychiatry, Psychology and Neuroscience, King’s College London, London, UK; 6 Department of Sociology and Center for Social Development and Education, University of Massachusetts Boston, Boston, USA; 7 Department of Child Psychiatry UPC, KU Leuven, Leuven, Belgium

**Keywords:** ASD, Parenting, Accommodation, Problem behaviour, Reactivity, Intolerance of Uncertainty, Extreme demand avoidance

## Abstract

Here, we explored the structure of the ‘Parenting Strategies Questionnaire’, a new scale designed to measure parenting strategies for problem behaviour in ASD. We then examined links between child behaviour and parenting in a sample of 222 predominantly-UK parents of ASD children exhibiting behaviour found difficult or challenging. Analysis revealed three parenting subscales:* Accommodation*,* Reinforcement Approaches* and* Reducing Uncertainty*. Both* Accommodation* and* Reducing Uncertainty* were linked to child problem behaviour. Child factors explained up to 29% of the variance in* Accommodation*, with* Socially Inflexible Non-compliance* the strongest predictor, and up to 24% of the variance in* Reducing Uncertainty*, with* Intolerance of Uncertainty* the strongest predictor. Child factors were not related to* Reinforcement Approaches*. Longitudinal studies investigating these relationships are needed.

**Electronic supplementary material:**

The online version of this article (10.1007/s10803-019-04219-2) contains supplementary material, which is available to authorized users.

Autism Spectrum Disorders (ASD) are neurodevelopmental impairments characterised by difficulties with communication, socialisation, and rigid and repetitive behaviours (Americal Psychiatric Association 2013). Problem behaviour (also referred to as ‘behaviour that challenges’ or, in the past, ‘challenging behaviour’) often occurs in children with ASD, and is more severe in ASD than in other clinical populations (e.g., Brereton et al. 2006; Estes et al. 2009). Forms of problem behaviour include oppositionality, failures to comply, destructiveness and explosiveness (e.g., Gadow et al. 2004). These behaviours are thought to reflect a dysregulated emotional state, resulting in outbursts and prolonged emotional reactions (Mazefsky et al. 2018a, b). Problem behaviour may reflect attempts by the child to reduce anxiety or distress by escaping aversive activities, or reactivity reflecting frustration when things are not on their terms (Brewer et al. 2014; Larson 2006).

Demands to comply have been identified as a key trigger of reactivity in ASD (Chowdhury et al. 2016). Some individuals appear more reactive to routine demands (e.g., to wash or get dressed), and others to demands in socially challenging or novel situations (e.g., when visiting friends) (Chowdhury et al. 2016). The former ‘demand-specific’ profile resembles accounts of extreme/‘pathological’ demand avoidance (‘PDA’), which describe avoidance of and reactivity to routine demands in children with ASD (Newson et al. 2003). Some accounts of PDA explicitly attribute these behaviours to elevated anxiety and distress in the context of demands (Newson et al. 2003). In contrast, the latter ‘socially inflexible’ profile, may particularly reflect intolerance of uncertainty: the tendency to “react negatively on an emotional, cognitive, and behavioural level to uncertain situations and events” (Buhr and Dugas 2009, p. 216), which characterizes some children with ASD (Boulter et al. 2014; Larson 2006).

## Parenting and Problem Behaviour in ASD

The demands on parents of children with ASD to manage high levels of reactivity and avoid excessive disruption to daily activities presents a major challenge. Qualitative and case studies report that parents spend considerable effort in making adaptations to regulate their child’s affect (e.g. by adjusting performance demands, limiting exposure to stressors) and avoid confrontation (e.g. by ensuring that the child’s preferred routines are followed), (O’Nions et al. 2018). This set of strategies has previously been termed ‘accommodation’ (Calvocoressi et al. 1999).

Although accommodation is not described in the general parenting literature (Power 2013), it has been well studied in the context of anxiety and OCD (e.g., Lebowitz et al. 2012). A recent questionnaire study reported daily accommodation of rigid and repetitive behaviours (RRBs) by more than half of a sample of 86 parents of ASD children aged 1–16 years (Feldman et al. 2019). Parents reported feeling compelled to accommodate in order to prevent aggressive outbursts. Findings indicated a robust positive relationship between severity of RRB symptoms and accommodation, similar to previous reports of links between child anxiety severity and parental accommodation in families of ASD children (Storch et al. 2015).

As well as accommodation, some studies suggest that parents attempt to use more traditional behavioural management approaches to reduce non-compliance or problem behaviour during daily activities. Qualitative accounts describe unsuccessful attempts to use negative reinforcement (e.g. punishment or time-out), resulting in the child maintaining or escalating problem behaviour (e.g., Agazzi et al. 2013; Armstrong et al. 2015; Lucyshyn et al. 2007). An observational study reported that, instead of punishing non-compliance during routine activities, parents of children with developmental disabilities responded with forbearance: providing assistance and reducing demands (Lucyshyn et al. 2004). Larger-scale studies also suggest that parents of ASD children typically allow more leeway with regards to rules and expectations. In a sample of 552 mothers of school-aged children with ASD, Maljaars et al. (2014) reported less use of rules and discipline, speculating that this may be due to a stronger focus on trying to prevent problem behaviour. In a sample of 30 mothers of school-aged children with ASD, Boonen et al. (2015) reported less ‘structuring’ by parents during interactions, compared to that seen in 39 typically-developing controls. The authors noted that children with ASD did not always comply with requests, and often pursued tasks in their own way, speculating that mothers may have learned not to intervene immediately to avoid evoking the child’s frustration. Therefore, on the whole, it appears that parents of children with ASD use rules, demands, and discipline to a lesser degree than matched typically-developing comparison groups.

Indeed, findings suggest that a less authoritative approach may help parents of children with ASD to co-regulate their emotions. Hirschler-Guttenberg et al. (2015) found that, in pre-schoolers with ASD, authoritative (strict/high warmth) parenting, which has been linked to better outcomes in typical development (e.g., Calders et al. 2019), was associated with less seeking of reassurance in a fear-evoking situation. Instead, mothers who were less authoritative appeared to optimise their child’s capacity to adaptively reduce fear through seeking parental support. In the context of a frustrating event, ‘authoritarian’ (strict/low warmth) parenting was linked to an increase in the child’s attempts to self-regulate, e.g. using avoidance and repetitive behaviour (Hirschler-Guttenberg et al. 2015). These findings suggest that parenting ASD children might disproportionately require sensitivity and warmth. Indeed, a recent multi-method study exploring parent–child interactions in 44 adolescents with ASD and 38 matched controls suggested that mothers of ASD adolescents showed more sensitivity and creativity when engaging their child in a structured task (van Esch et al. 2018).

Cross-sectional and longitudinal studies have also explored the question of whether problem behaviour in ASD is linked to use of discipline and control strategies. Maljaars et al. (2014) reported a modest cross-sectional association between the severity of problem behaviour and parental control strategies, including rules and discipline, with a larger association in the ASD sample (*n* = 552) compared to the typically-developing comparison group (*n* = 437). Boonen et al. (2014) reported that negative control (discipline and harsh punishment) was a cross-sectional predictor of child problem behaviour in a sample of 206 children with ASD, taking into account other child-factors. Both negative forms of parental control and poorer ‘limit setting’ (e.g. sometimes losing one’s temper, sometimes giving in to the child) have also been found to predict increases in problem behaviour in children and adolescents/young adults with ASD over time (Dieleman et al. 2017; Osborne et al. 2008). Therefore, it appears that where parents do adopt negative forms of discipline and control, these may be associated with poorer outcomes.

## The Present Study

There is a pressing need to better understand the relationships between parenting strategies and child problem behaviour in families of children with ASD, in particular, accommodation, which has been relatively neglected in the literature to date. Furthermore, existing studies measuring accommodation refer to accommodation of specific symptom domains, and do not provide specific exemplars of concrete parenting behaviours that are recognizable for parents of children with ASD. Therefore, it is necessary to develop a measure that captures manifestations of accommodation (and other parenting strategies) as they occur in everyday life in families of children with ASD.

Recently, we developed a new scale called the Parenting Strategies Questionnaire, to quantify behaviours relevant to managing problem behaviour in children with ASD. Items were based on themes derived from a meta-synthesis of naturally-occurring parenting behaviours to manage irritability, challenging behaviour, non-compliance and anxiety in ASD, drawing on accounts of observational, qualitative and case studies (O’Nions et al. 2018). The new measure included all 45 themes identified, and therefore was not designed along a particular theoretical framework.

In the present paper, our first aim is to investigate the structure of the Parenting Strategies Questionnaire (PSQ), and explore relationships between the resulting dimensions and parenting behaviours captured using existing measures: the Parenting Behaviour Scale-ASD version (PBS, Lambrechts et al. 2011, Maljaars et al. 2014), and the Alabama Parenting Questionnaire (APQ, Essau et al. 2006; Frick et al. 1999). These measures were chosen to cover (a) general parenting dimensions (e.g. positive reinforcement, parental involvement, discipline, rules), (b) ASD-adapted parenting (e.g. stimulating development, adapting the environment), and (c) parenting implicated in the development of problem behaviour in non-ASD populations (e.g. inconsistent or harsh discipline, poor monitoring and supervision).

Our second aim is to investigate cross-sectional associations between parenting strategies and child characteristics, to identify whether particular child problem behaviour-related variables are differentially associated with parenting strategies. Our final aim is to explore the extent to which child problem behaviour severity and other child factors predicted use of different parenting strategies using regression modelling in cross-sectional data.

Since no studies have yet explored links between accommodation and problem behaviour in ASD, we were unable to generate firm hypotheses, and our analyses were therefore exploratory and hypothesis generating. Indeed, despite the apparent dissimilarity between these approaches, based on the extant literature, child problem behaviour might be cross-sectionally linked to both more authoritarian and more accommodative parenting behaviours (e.g., Boonen et al. 2015; O’Nions et al. 2018).

## Methods

### Participants

The current study was undertaken as part of an ongoing longitudinal study investigating parenting strategies and child behaviour. The study was approved by the KU Leuven Societal and Public Ethics Committee. Informed consent was obtained from all individual participants included in the study. The full study sample (393 parents/caregivers) was recruited via electronic links posted on social networks by the research team and, at the request of the research team, by other organisations for parents of children with ASD and/or other neurodevelopmental profiles or disabilities, including extreme/‘pathological’ demand avoidance. A snowballing strategy was used such that parents who had participated were encouraged to share information about the study with others to facilitate recruitment.

The present analysis uses data collected from 222 parents/caregivers of children aged between 6 and 17 years, who took part in the first wave of data collection, for whom data on both child behaviour and parenting were available. Inclusion criteria for the present analysis were that (a) the parent/caregiver was living with the child full-time or part-time; (b) the parent/caregiver reported that the child had a diagnosis of ASD; (c) the child’s score on the Social Communication Questionnaire (SCQ; Rutter et al. 2003) was 12 or above (as per Mazefsky et al. 2018b); and (d) that the child was reportedly displaying difficult or challenging behaviours within the last 6 months.

### Procedure

Data were gathered through self-administered questionnaires collected electronically, completed by the child’s parent/caregiver. Parents of any child within the age range were invited to take part, and recruitment materials highlighted that we were keen to recruit parents of children exhibiting a range of behaviours and profiles. Questionnaires included items on child, parent/caregiver, and family characteristics, and other issues. Analyses were conducted using STATA (Release 16; StataCorp, College Station TX).

### Sample Characteristics

Data were collected on background family characteristics, including parent/caregiver age, number of children in the family, parent/caregiver (and cohabiting partner’s) educational qualifications, parent/caregiver (and cohabiting partner’s) employment. Parents/caregivers also provided information about their child (e.g. their age, diagnoses and educational placement, estimated academic level relative to mainstream peers, and degree of independence in daily living tasks). We generated a proxy indicator of SES within the present sample by combining information on educational qualifications and employment, using the Office for National Statistics coding tool (Office for National Statistics UK, n.d.). Data on SES were calculable for n = 185.

Mean age of the children on whom parents/caregivers reported was 11 years 1 month (range 6 years 1 month–16 years 8 months), and the median age band of parent/caregiver respondents was 40–44 years. Other details of the sample are provided in Table 1.

**Table 1:** Sample characteristics

	N (%)
Child characteristics	
Male	156 (70)
Female	64 (29)
Complex gender identity	2 (1)
Reported diagnoses	
ASD	222 (100)
Anxiety	68 (31)
ADHD	61 (27)
PDA/demand avoidance	75 (34)
Mood disorder or major depression	17 (8)
Conduct problems/ODD/challenging behaviour	15 (7)
Mild intellectual disability	23 (10)
Moderate intellectual disability	25 (11)
Severe intellectual disability	13 (6)
Reported use of language to communicate	
Mostly verbal	217 (98)
Mostly non-verbal	5 (2)
Reported academic level	
Similar to/ahead of mainstream peers	73 (33)
Similar to mainstream peers apart from specific difficulties	58 (26)
Slightly behind mainstream peers	15 (7)
Markedly behind peers	44 (20)
Very far behind mainstream peers	32 (14)
Reported independence in daily living skills (e.g. dressing, washing)	
Similar to or ahead of others of their age	38 (17)
Need a bit more help than others their age	60 (27)
Need a lot more help than others their age	99 (45)
Completely dependent on parental/carer assistance	25 (11)
Child educational setting	
Mainstream school without extra help	25 (11)
Mainstream/unit within mainstream school with extra help	77 (35)
Specialist or alternative setting	74 (33)
Home-educated	33 (15)
Left school or not currently enrolled in school	13 (6)
Parent/caregiver characteristics	
Female gender	214 (96)
Residing in the UK	220 (99)
Child’s natural parent	210 (95)
Married/cohabiting	162 (73)
White European ethnicity	217 (98)
Post school-age qualifications^b^	150 (68)

^a^Data on diagnoses were updated based on new diagnoses received within the 12-month window after study participation

^b^Higher National Certificate, a Higher National Diploma or an undergraduate degree (i.e. post school-age qualifications)

Parents with an interest in extreme/‘pathological’ demand avoidance (PDA) were highly represented in the sample, with 116 (52%) mentioning extreme or pathological demand avoidance or demand avoidance behaviour, as part of their child’s diagnostic description, a profile that they believed might fit their child, or a concept that had informed their parenting approach. Given the nature of the snowballing recruitment strategy, an even greater proportion of respondents may be familiar with the PDA concept and the associated formulation of avoidance in ASD being associated with anxiety.

### Measures

Measures analysed in the present study are described in detail below. Total scores for scales and subscales were calculated by taking the mean of the items multiplied by the number of items for which data were available, provided that data for at least 50% of items were present.

### Parenting Strategies to Manage Problem Behaviour

Parenting strategies to manage problem behaviour and emotional reactivity were assessed using the newly developed PSQ. The PSQ items were developed to capture the 45 subthemes identified in our previous meta-synthesis (O’Nions et al. 2018). Modifications to subtheme titles were made by the first and last author to improve the concreteness of questionnaire items. Responses for PSQ items were made on a 5-point scale (0 = Never, 1 = Almost never, 2 = Sometimes, 3 = Often, 4 = Always) to index how frequently respondents employed the 45 different behaviours when parenting their child with ASD. Dimension reduction using principal component analysis (PCA) with a Varimax rotation was used to identify subscales prior to analyses (see “Results” section for details).

### Other Parenting Measures

Parenting behaviours were measured using the *Parenting Behaviour Scale*-*ASD version* (Lambrechts et al. 2011; Maljaars et al. 2014). This scale consists of five parenting dimensions designed to capture general population individual differences, and two that are considered ‘ASD adapted’. Responses to items are made on a 5-point scale (1 = Never/Almost never, 2 = Rarely, 3 = Sometimes, 4 = Often, 5 = Always). In order to give the scale a minimum possible score of 0, we recoded items so that ‘Never/ Almost never’ received a score of 0.

An exploratory factor analysis conducted in a separate dataset (*n* = 862 parents of children aged 6–16 years (mean = 11.12, SD = 2.49), *n* with ASD = 509) was used to reduce the number of items collected for this scale (information available from the authors on request). Based on these results, we collected only 8 (out of 11) items from the original *Positive Parenting* subscale (e.g. “When my child has a problem, we look at different possible solutions together”), six (all) from the *Discipline* subscale (e.g. “When my child does something that is not allowed, I give him/her a punishment”), and four (out of 6) from the *Rules* subscale (e.g. “I make agreements with my child about how he/she should behave”), plus 10 (out of 11) from the *Stimulating Development* (ASD adapted; e.g., “When someone is crying, I explain to my child what that person is feeling and why”), and six (out of 9) from *Adapting the Environment* (ASD adapted; e.g. “I make sure that my child is not overstimulated”). Correlations with the full subscales calculated in the existing dataset on which exploratory PCA was conducted were *r*  = .96 for *Positive Parenting*, *r* = .93 for *Rules*, *r* = .99 for *Stimulating Development*, and *r* = .95 for *Adapting the Environment*. Since we collected 50% or fewer items for the other published subscales (*Material Rewarding* and *Harsh Punishment*), total scores were not calculated. Internal reliabilities were acceptable, at >.7 for all subscales, apart from *Adapting the Environment* (Table 2).

**Table 2:** Information on scales that formed part of the present analyses

	n	No. items	Relia- bility (alpha)	Total score			Item score		
				Mean	SD	Range	Mean	SD	Range
Parenting measures									
Discipline (PBS)	222	6	.84	7.52	4.90	0–19	1.25	.82	0–3.2
Rules (PBS)	222	4	.81	9.85	3.84	0–16	2.46	.96	0–4
Positive parenting (PBS)	222	8	.72	25.06	4.32	13–32	3.13	.54	1.6–4
Stimulating development (PBS)	222	10	.84	28.31	6.07	12–40	2.83	.61	1.2–4
Adapting the environment (PBS)	222	6	.58	14.20	3.99	3–24	2.37	.66	0.5–4
Parental involvement (APQ)	222	10	.72	26.42	6.70	7–40	2.64	.67	0.7–4
Positive parenting (APQ)	222	6	.84	18.51	4.05	5–24	3.09	.67	0.8–4
Poor supervision (APQ)	221	10	.67	2.37	3.18	0–18	.24	.32	0–1.8
Inconsistent discipline (APQ)	222	6	.65	7.47	3.68	0–17	1.25	.61	0–2.8
Physical punishment (APQ)	222	3	.91^a^	.27	.76	0–4	.09	.25	0–1.3
Child measures									
ASD symptoms (SCQ)	221	39	.80	24.12	6.18	12–38	.62	.16	0.3–1
Reactivity (EDI)	222	24	.97	61.48	21.91	7–96	2.56	.91	0.3–4
Demand-specific (HSQ)	222	12	.90	66.07	23.72	3–106	5.51	1.98	0.3–8.8
Socially inflexible (HSQ)	221	12	.88	69.34	21.50	6–108	5.78	1.79	0.5–9
Extreme demand avoidance (EDA)	221	20	.91	47.81	13.37	4–68	2.08	.58	0.2–3
Intolerance of uncertainty (IU)	222	12	.89	33.76	9.7	6–48	2.81	.81	0.5–4

^a^Reliability coefficient based on two items, due to absence of variance on one item

Parenting behaviours were also measured using the *Alabama Parenting Questionnaire* (APQ; Essau et al. 2006; Frick et al. 1999). The APQ is designed to measure dimensions of parenting that are relevant to the development of child conduct problems. The 42 items are rated on a 5-point scale (1 = Never, 2 = Almost never, 3 = Sometimes, 4 = Often, 5 = Always). In order to give the scale a minimum possible score of 0, we recoded these so that ‘Never’ received a score of 0, and ‘Always’ received a score of 4. APQ subscales are *Parental Involvement* (10 items; e.g. “You have a friendly talk with your child”), *Positive Parenting* (6 items; e.g. “You reward or give something extra to your child for obeying you or behaving well”), *Poor Supervision/Monitoring* (10 items; e.g. “You get so busy that you forget where your child is and what he/she is doing”), *Inconsistent Discipline* (6 items; e.g. “You threaten to punish your child and then do not actually punish him/her”), and *Physical Punishment* (3 items; e.g. “You slap your child when he/she has done something wrong”).

### Child ASD Severity

Child ASD severity was measured using the *Social Communication Questionnaire (SCQ)*—*Lifetime Version* (Rutter et al. 2003). The SCQ is a 40-item parent-report screening measure designed to quantify behaviours associated with ASD, which was originally based on the Autism Diagnostic Interview—Revised (Lord et al. 1994). Parents are asked to respond “Yes” or “No” to each of the items. Nineteen items focus on the entire developmental history, and 21 on the child’s behaviour when he/she was aged 4–5 years old. As described in the manual (Rutter et al. 2003), only 39 of the 40 items contribute to the total score, indexing the child’s ASD severity.

### Child Problem Behaviour-Related Measures

The *Emotion Dysregulation Inventory (EDI)* was used to quantify observable signs of emotional dysregulation (Mazefsky et al. 2018a, b). The measure consists of 30 items and has two subscales: *Reactivity* (24 items) e.g. high arousal, aggression, emotional outbursts, rapid escalation in intensity, extreme emotional responses, and *Dysphoria* (6 items), e.g. lower arousal, unease, anxiety and low mood. Items are rated on a 5-point thermometer scale (0 = Not at all, 1 = Mild, 2 = Moderate, 3 = Severe, 4 = Very severe), with severity capturing both frequency and intensity. A recent study suggested excellent reliability, validity and psychometric properties, and greater sensitivity compared to measures of externalising and internalising problem behaviour in participants with ASD (Mazefsky et al. 2018b). Only data on *Reactivity* were analysed in the present study.

Context-sensitive manifestations of non-compliance and reactivity were measured using the *Home Situations Questionnaire*—*ASD* (HSQ-ASD, Chowdhury et al. 2010, 2016). Severity scores reflect the intensity of reactivity and problem behaviour when faced with instructions, commands or rules, with *Demand*-*Specific Non*-*compliance* items describing reactivity in routine contexts, such as getting up, getting dressed etc., and *Socially Inflexible Non*-*compliance* items describing reactivity in less routine and social situations, e.g. when visiting friends. The HSQ-ASD has a range of 0–9 for the 24 constituent items (a score of 0 designating no problems, 1–3 designating “Mild”, 4–6 designating “Moderate” and 7–9 designating “Severe” problems). Each subscale consists of 12 items.

Child extreme demand avoidance was measured using the *Extreme Demand Avoidance Questionnaire* (EDA-Q; O’Nions et al. 2014). The EDA-Q consists of 26 items describing the characteristics associated with extreme/’pathological’ demand avoidance (e.g. resisting ordinary demands and requests, being driven by the need to be in charge, meltdowns if pressurised to do something). Items are rated on a four-point scale (0 = Not at all true; 1 = Some-what true, 2 = Mostly true, 3 = Very true). This analysis employed a reduced version of this scale identified using PCA (O’Nions et al., in prep), which included 23 out of the original 26 items, forming a single subscale.

Child intolerance of uncertainty was measured using the *Intolerance of Uncertainty Scale* (IU) (Carleton et al. 2007). The IU consists of 12 parent-report items designed to measure anxiety in response to uncertainty in the environment (e.g. when things happen suddenly, when the child feels unsure of what to do). Items are rated on a five point scale (1 = Not at all like them, 3 = Moderately like them, 5 = Entirely like them). In order to give the scale a minimum possible score of 0, we recoded these so that ‘Not at all like them’ received a score of 0, and ‘Entirely like them’ a score of 4.

Details on the number of participants for whom data were available on each measure, the scale reliabilities, means, standard deviations and ranges are provided in Table 2.

## Results

### Descriptive Characteristics of the Sample

Characteristics of the present sample indicate very high levels of non-compliance and reactivity. Comparisons with US general and ASD population norms indicate that 92% of the present sample showed clinically elevated levels of *Reactivity* relative to the general population, and 47% scored one standard deviation or higher than the ASD sample norm (Maljaars et al. 2014). In addition, 62% of the sample scored at least one standard deviation above the mean for* Demand Specific Non-compliance*, and 55% scored one standard deviation above the mean for* Socially Inflexible Non-compliance* based on a pooled sample derived from two intervention studies in children with ASD and co-occurring irritability/hyperactivity (Chowdhury et al. 2016).

### Aim 1: Exploring the Structure of the Parenting Strategies Questionnaire and Relationships with Other Parenting Dimensions

Following Hastings et al. (2005) and Benson (2010), a PCA with Varimax rotation was used to explore patterns of covariance among PSQ items. One item (“Smack the child”) was removed from the analysis, since it was endorsed at extremely low rates (97% of the sample indicated ‘Never’ or ‘Almost Never’). Examination of the scree plot (Cattell 1966) was used to inform the extraction of the optimal number of subscales. The scree plot showed an elbow at the fourth eigenvalue, so PCA was repeated with the number of extracted components constrained to three, which collectively explained 35% of the measured variance.

To explore the reliability of the identified structure, the sample was split randomly, and the PCA repeated. Tucker’s congruence coefficients between the loadings of the subsamples (after Procrustes rotation towards the loadings of the overall sample) were >.95 for all but the third component in subsample 2 (subsample 1, values: .97, .98, .96; subsample 2, values: .97, .97, .92 for each of the three components respectively). Based on work by Lorenzo-Seva and Ten Berge (2006) congruence coefficients >.95 imply that the corresponding components can be interpreted as identical.

The results of the PCA conducted in the full sample were used to inform the inclusion of items in relevant subscales. Applying cut-offs used by Benson (2010), items were retained in a subscale if they loaded ≥ .|40| on one of the components and (2) < .|40| onto any other components. Based on these criteria, 31 of the total 45 items loaded exclusively onto one of the three components. Details of the resulting measure are presented in Table 3.

**Table 3:**
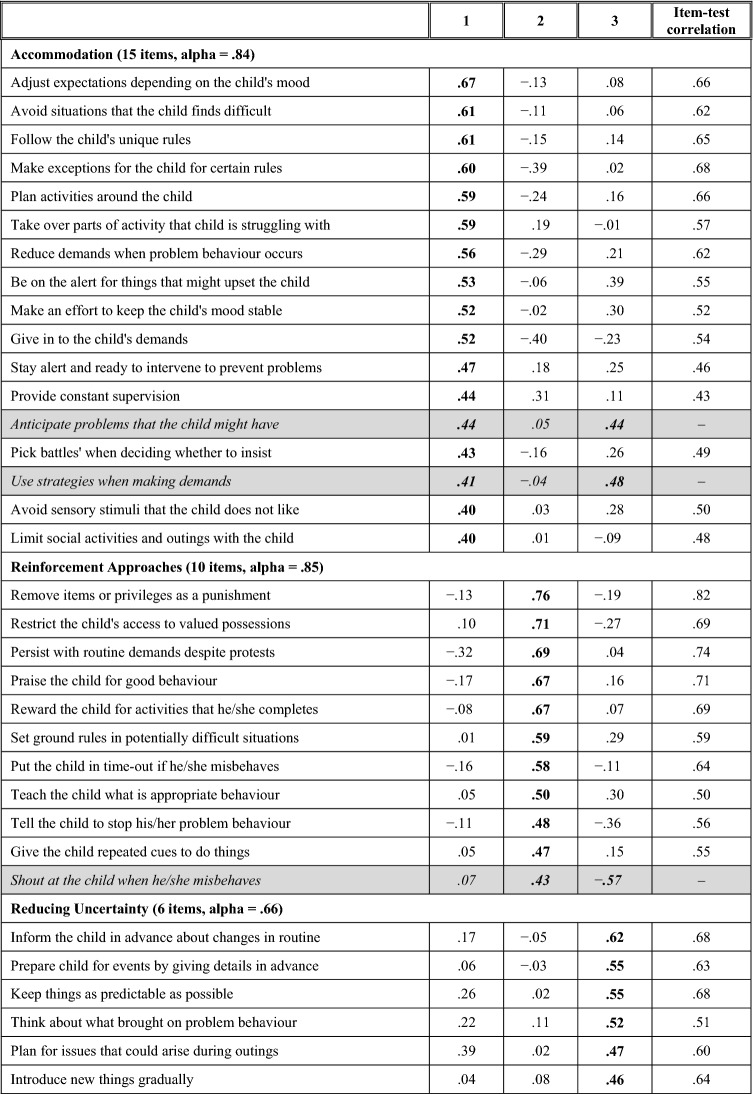
Abbreviated versions of Parenting Strategies Questionnaire items and their loadings onto extracted principal components

Items that are italicised and highlighted in grey were not included in the associated subscale due to loadings on more than one component. Loadings ≥.|40| are presented in bold

The first subscale, consisting of 15 items, was termed ‘*Accommodation*’. It included behaviours such as adjusting expectations depending on the child’s mood, avoiding situations that the child finds difficult, and following the child’s unique rules for how things should be done. The second subscale, consisting of ten items, was termed ‘*Reinforcement Approaches’*. Items described use of rules and contingent rewards/punishments to incentivise compliance. The third subscale, consisting of six items, was termed ‘*Reducing Uncertainty’.* It described use of advance preparation and warning for upcoming events, maintaining predictability, and thinking about the triggers of problematic episodes.

Total scores for PSQ subscales were calculated using the same approach as that adopted with our other sample measures (taking the mean of the items and multiplying by the number of items in the scale). Mean subscale scores were 45.91 for *Accommodation* (SD = 6.69, range 26–60, mean item score = 3.06), 22.59 for *Reinforcement Approaches* (SD = 6.87, range 6–37.78, mean item score = 2.26), and 19.50 for *Reducing Uncertainty* (SD = 2.80, range 9–24, mean item score = 3.25). Examination of relationships between subscales revealed a negative association between *Accommodation* and *Reinforcement Approaches* (*r *= − .26, p < .001), and a positive association between *Accommodation* and *Reducing Uncertainty* (*r *= .43, p < .001). No significant association was detected between *Reinforcement Approaches* and *Reducing Uncertainty* in this sample (*r *= − .01, p > .1). Since *Reducing Uncertainty* showed negative skew, we repeated correlation analysis using Spearman’s correlations, which produced almost identical results (differences between Pearson and Spearman correlation coefficients ≤ .02).

As our final step in pursuing the first aim of the study, we explored associations between PSQ subscales and other parenting measures. One subscale (*APQ Physical Punishment*) was dropped from further analysis due to only 14% of subjects scoring above 0. Pearson correlation coefficients describing the relationships between each of the PSQ subscales and other parenting dimensions are presented in Table 4, with results surviving Bonferroni correction presented in bold. Given the negative skew for *Reducing Uncertainty* and *APQ Positive Parenting*, and the positive skew for *APQ Poor Supervision*, analyses were repeated using Spearman’s rank correlations for these variables. The pattern of associations was very similar (differences in correlation coefficients ≤ .04).

**Table 4:** Pearson correlation coefficients describing relationships between Parenting Strategies Questionnaire subscales and other parenting dimensions

	Accommodation	Reinforcement Approaches	Reducing Uncertainty
Parenting Behaviour Scale (PBS)			
PBS Discipline	− .36***	.73***	− .09
PBS Rules	− .19**	.59***	.17**
PBS Positive Parenting	.03	.30***	.30***
PBS Stimulating Development	.08	.24***	.31***
PBS Adapting the Environment	.08	.43***	.42***
Alabama Parenting Scale (APQ)			
APQ Parental Involvement	− .09	.39***	.13*
APQ Positive Parenting	− .15*	.63***	.00
APQ Poor Supervision	− .19**	.02	− .16*
APQ Inconsistent Discipline	.05	.12	− .22***

Bonferroni adjustment indicates an alpha threshold of p<.0019 (27 correlations calculated). Findings that survive Bonferroni correction within models are presented in bold

*p < .05, **p < .01, ***p < .001

Considering associations with existing parenting measures, *Accommodation* was linked to lower scores for *PBS Discipline* (and, at a nominal threshold, to lower scores for *PBS Rules*, *APQ Positive Parenting*, and *APQ Poor Supervision*). No other existing parenting scales were significantly related to amount of *Accommodation*. In contrast, *Reinforcement Approaches* were positively related to all PBS subscales, most strongly to *PBS Discipline*. *Reinforcement Approaches* were also positively linked to *APQ Positive Parenting* and *APQ Parental Involvement*. The stronger association between *Reinforcement Approaches* and *APQ Positive Parenting* compared to *PBS Positive Parenting* may reflect the focus of the former on contingent positive reinforcement, whilst the latter describes non-contingent positive parental engagement.

For *Reducing Uncertainty*, modest to moderate positive associations were found with *PBS Positive Parenting, PBS Stimulating Development* and *PBS Adapting the Environment*. In addition, *Reducing Uncertainty* was negatively related to *APQ Inconsistent Discipline*.

### Aim 2: Exploring Links Between Parenting Strategies and Child Behavioural Dimensions

The second aim of the study was to investigate cross-sectional associations between parenting strategies and child characteristics, to identify whether particular child characteristics and problem behaviour-related variables are differentially associated with parenting strategies. We explored links between PSQ dimensions and child-level variables (background factors, ASD characteristics, and child problem behaviours; Table 5). Since parental demographic factors (age, SES, educational level, family size) showed no associations with PSQ subscales (rs < |.1|), no adjustments were made for these factors in subsequent analyses.

**Table 5:** Spearman correlation coefficients depicting relationships between Parenting Strategies Questionnaire subscales and child demographic, background, and child problem behaviour-related variables

	Accommodation	Reinforcement Approaches	Reducing Uncertainty
(A) Child demographic and background factors			
Child age	− .12	− .07	.09
Child gender	.01	− .07	.13
Child lower academic level	.16*	.14*	.01
Child lack of independence in daily living	.30***	− .02	.14*
Child ASD severity (SCQ)	.12	.16*	.23***
(B) Child problem behaviour-related variables			
Reactivity (EDI)	.50***	.02	.20**
Demand-Specific (HSQ)	.41***	− .03	.13
Socially Inflexible (HSQ)	.53***	− .05	.22**
Extreme Demand Avoidance (EDA)	.44***	− .13*	.15*
Intolerance of Uncertainty (IU)	.29***	− .16*	.48***

Bonferroni adjustment across Parenting Strategies Questionnaire subscales and child factors indicates an alpha threshold of p < .0017 (30 correlations calculated). Findings that survive Bonferroni correction are presented in bold

*p < .05, **p < .01, ***p < .001

For child-relevant variables that were not continuous, Spearman’s rank correlation coefficients are presented, and for continuous variables, Pearson’s correlation coefficients, with results surviving Bonferroni correction presented in bold. Because some measures (*Reducing Uncertainty, Extreme Demand Avoidance* and *Intolerance of Uncertainty*) showed negative skew, Spearman’s rank correlations were also calculated and compared with the Pearson’s estimates. Differences between Pearson and Spearman’s correlation coefficients were ≤ .04 for all but one association: that between* Reducing Uncertainty* and* Intolerance of Uncertainty* (*r *= .47, *r*_s_= .41).

Correlation analysis revealed that degree of *Accommodation* was robustly related to child problem behaviour-related variables—with more severe difficulties on each of the variables linked to greater *Accommodation*. The strongest association was between *Accommodation* and *Socially Inflexible Non*-*compliance* (a subscale of the HSQ), whilst the weakest was with *Intolerance of Uncertainty*. Child lack of independence in daily living skills was also associated with greater *Accommodation* at the Bonferroni-adjusted threshold. Child problem behaviour-related dimensions were not associated with *Reinforcement Approaches* in this sample. However, *Reducing Uncertainty* was associated with ASD severity, *Socially Inflexible Non*-*compliance* and *Intolerance of Uncertainty*—with more *Reducing Uncertainty* associated with higher scores on these measures.

### Aim 3: Exploring the Extent to Which Child Factors Predict Use of Different Parenting Strategies

To pursue our final aim, we conducted regression analysis to identify which child factors were independent predictors of parenting strategies. With the exception of child gender, all dependent and independent variables were standardised, such that the beta values are directly comparable within and between models. We ran separate regression models for each child problem behaviour variable and each parenting strategy, with other child variables held constant across models. Findings are presented in Table 6.

**Table 6:** Regression models predicting parenting dimensions from child demographic factors and child problem behaviour-related variables

	Standardised regression coefficients for models containing each child problem behaviour-related variable				
	(1)	(2)	(3)	(4)	(5)
(A) Models predicting Accommodation					
Child age	− .06	− .13*	− .08	− .08	− .19**
Child gender	− .02	.06	.09	.03	.00
Child lower academic level	.07	.08	.05	.12	.15*
Child lack of independent living skills	.22***	.14	.14*	.22***	.23***
Child ASD severity (SCQ)	− .05	.00	− .04	.02	− .05
(1) Reactivity (EDI)	.46***				
(2) Demand-Specific (HSQ)		.33***			
(3) Socially Inflexible (HSQ)			.47***		
(4) Extreme Demand Avoidance (EDA)				.42***	
(5) Intolerance of Uncertainty (IU)					.33***
*Variance in parenting explained*	.29***	.19***	.29***	.26***	.19***
(B) Models predicting Reinforcement Approaches					
Child age	− .08	− .08	− .08	− .09	− .06
Child gender	− .05	− .04	− .03	− .02	.00
Child lower academic level	.11	.11	.10	.09	.08
Child lack of independent living skills	− .13	− .12	− .10	− .11	− .10
Child ASD severity (SCQ)	.18*	.18*	.18*	.17*	.20*
(1) Reactivity (EDI)	.00				
(2) Demand-Specific (HSQ)		− .03			
(3) Socially inflexible (HSQ)			− .09		
(4) Extreme Demand Avoidance (EDA)				− .12	
(5) Intolerance of Uncertainty (IU)					− .13
*Variance in parenting explained*	***.02***	***.02***	***.03***	.04*	.04*
(C) Model predicting Reducing Uncertainty					
Child age	.10	.07	.08	.08	.00
Child gender	.25	.28*	.28*	.27*	.15
Child lower academic level	− .11	− .11	− .11	− .09	− .01
Child lack of independent living skills	.10	.08	.05	.11	.05
Child ASD severity (SCQ)	.22**	.24**	.22**	.24***	.18**
(1) Reactivity (EDI)	.17**				
(2) Demand-Specific (HSQ)		.09			
(3) Socially Inflexible (HSQ)			.21**		
(4) Extreme Demand Avoidance (EDA)				.14*	
(5) Intolerance of Uncertainty (IU)					.42***
*Variance in parenting explained*	.10***	.08***	.11***	.09***	.24***

Separate regression models were run for each child problem behaviour variable and each parenting strategy on standardized variables (gender not standardised). Model (1) contained Reactivity as the child problem behaviour-related variable, (2) Demand-Specific, (3) Socially Inflexible, (4) Extreme Demand Avoidance, (5) Intolerance of Uncertainty

Bonferroni adjustment for the overall significance of the regression models indicates an alpha threshold of p < .003 (15 models estimated). Bonferroni correction within models containing six independent predictors indicates an alpha threshold of p < .008. Findings that survive Bonferroni correction within models are presented in bold. The proportion of variance in parenting explained is presented in bold italics for models that survive Bonferroni-adjusted significance thresholds

*p < .05, **p < .01, ***p < .001

Regardless of the child problem behaviour variable included, all models predicting *Accommodation* from child-factors were significant at the Bonferroni-corrected threshold. The proportion of variance in parenting explained ranged from 19 to 29%, depending on the child problem behaviour variable included.

Across all models, the strongest predictor of *Accommodation* was the severity of the child problem behaviour measure. For all but one model (that containing *Demand*-*Specific Non*-*compliance*), child lack of independence in daily living skills was also a significant predictor. For the model containing *Intolerance of Uncertainty*, age was also a negative predictor, with less *Accommodation* at older ages.

In line with the correlation analysis, child factors did not significantly predict variance in *Reinforcement Approaches*, explaining only 2–4% of the variance. Within models, no child-level predictors were significant at corrected thresholds. In contrast, all models predicting *Reducing Uncertainty* from child factors were significant at Bonferroni-corrected thresholds, with 8–24% of the variance explained by modelled variables. Greater child ASD severity was associated with more *Reducing Uncertainty* across all models. In addition, for models containing *Socially Inflexible Non*-*compliance* and *Intolerance of Uncertainty* as the child problem behaviour variable, this was also a significant predictor.

### Exploratory Analysis: Investigating Links Between Parental Discipline and Child Problem Behaviour

Given that the newly developed PSQ does not contain a subscale specifically focusing on discipline/ negative reinforcement, we conducted an exploratory analysis using the *PBS Rules*, *PBS Discipline*, and *APQ Inconsistent Discipline* subscales to explore relationships with child factors in our sample. Findings are presented in full in Supplementary Tables 1 and 2, and summarised below.

In terms of child demographic and background factors, only two associations with discipline-related scales survived correction. First, child ASD severity was positively associated with *PBS Rules* (*r *= .22). Secondly, *Demand*-*Specific Non*-*compliance* and *Extreme Demand Avoidance* were positively related to *APQ Inconsistent Discipline* at the Bonferroni-adjusted threshold (*r *= .22 and .24, respectively; Supplementary Table 1).

We then conducted regression analysis to identify which child factors were independent predictors of discipline-related subscales (Supplementary Table 2), using the same approach as for the PSQ subscales. Although child-level factors as a whole did not significantly predict variation in *PBS Rules* at Bonferroni-adjusted thresholds (modelled variables explained only 4–5% of the variance), child ASD severity was a significant predictor across models, with greater ASD severity associated with higher scores for *PBS Rules.* Models predicting *PBS Discipline* were all non-significant, with only 1–3% of the variance in parenting explained by the modelled variables. Finally, for *APQ Inconsistent Discipline*, modelled variables explained 2–8% of the variance, with two models reaching Bonferroni adjusted thresholds for significance. In these models, the child behaviour variable (*Demand*-*Specific Non*-*compliance* and *Extreme Demand Avoidance*) was the only significant positive predictor of *APQ Inconsistent Discipline.*

## Discussion

The first aim of this study was to examine the component structure of a new questionnaire designed to quantify everyday parenting strategies to manage problem behaviour in ASD. Principal components analysis suggested that three underlying components best described variance on this measure. The first was termed ‘*Accommodation*’, and included making adjustments for the child, allowing things to be done on the child’s terms, and avoiding problematic situations or triggers. This subscale captures ways that parents manage their child’s environment, presumably with the goal of regulating their affect and preventing escalation of reactivity and problem behaviour. It is therefore very consistent with accounts of accommodation in non-ASD populations (Lebowitz et al. 2012).

Responses indicated that Accommodation was often used in our sample. Examining relationships between *Accommodation* and existing parenting measures, we detected a negative association with *PBS Discipline* that survived Bonferroni correction, suggesting that these two strategies do not usually co-occur. Surprisingly, no associations were found with the two ASD-oriented dimensions *PBS Stimulating the Development,* and *PBS Adapting the Environment*. Therefore, it appears that *Accommodation* is not well covered by the other parenting measures used here. Indeed, although the *PBS Stimulating the Development* and *PBS Adapting the Environment* are ‘ASD adapted’ measures, at an item level, they focus more on pedagogical approaches to enhance social development and autonomy (*Stimulating the Development*) and provision of routine, structure, contingent reinforcement, and management of distraction/ stimulation (*Adapting the Environment*).

The second PSQ subscale, ‘*Reinforcement Approaches’*, included items describing use of rules and contingent rewards/punishments as incentives for compliance. Items covered by *Reinforcement Approaches* resemble traditional behavioural management strategies applied in the context of typical development. *Reinforcement Approaches* were endorsed slightly less than *Accommodation* strategies in the present sample, and were modestly negatively related to *Accommodation*. Although negatively related, these approaches were not mutually exclusive, suggesting that parents may adopt either to some degree depending on the particular context.

Endorsement of *Reinforcement Approaches* was positively correlated with many existing parenting dimensions, most strongly with *PBS Discipline*, *PBS Rules* and *APQ Positive Parenting* (which focuses on contingent praise/reward). Interestingly, the *Reinforcement Approaches* subscale subsumes rewards/positive reinforcement and punishment/negative reinforcement, unlike other existing measures.

The third subscale, ‘*Reducing Uncertainty’,* included items describing strategies to maintain predictability, including advance preparation for and warning about upcoming events, and thinking about the triggers of problematic episodes. From a theoretical view point, *Reducing Uncertainty* could be seen as a form of accommodation, albeit one that is proactive: occurring in advance of potentially problematic episodes. To the best of our knowledge, this is the first full questionnaire subscale to focus on these strategies specifically.

Mean item endorsement suggested that *Reducing Uncertainty* was very commonly used in our sample, with the highest mean rating of any subscale. *Reducing Uncertainty* was moderately correlated with *Accommodation*, but not at all with *Reinforcement Approaches*. Examining links with existing subscales, we found significant associations between *Reducing Uncertainty* and *PBS Positive Parenting*, *PBS Stimulating the Development*, and *PBS Adapting the Environment*, plus a negative association with *APQ Inconsistent Discipline* surviving Bonferroni correction. Therefore, this subscale appears to have modest links with existing ASD-oriented and positive parenting dimensions, but in terms of content, is not well covered by existing measures.

### Links Between Parenting Strategies and Child Behaviour Variables

In the second part of the analysis, we explored the links between PSQ subscales and child problem behaviour-related variables, taking into account other child factors. We then conducted regression analysis to explore the relative contribution of child factors to variance on parenting behaviours. In regression analyses, child problem behaviour was the strongest contributor to variance in *Accommodation*, with the largest beta estimates obtained for *Reactivity, Socially Inflexible Non*-*compliance* and *Extreme Demand Avoidance*. Collectively, child factors explained up to 29% of the variance in *Accommodation*. This is consistent with qualitative reports that parents make accommodations to reduce disruption that might otherwise compromise family functioning (e.g., Lucyshyn et al. 2004), or ‘walk on eggshells’ to manage their child’s reactivity (Sabapathy et al. 2016). These findings are also in line with cross-sectional reports linking accommodation of RRBs or anxiety to symptom severity in these domains (Feldman et al. 2019; Storch et al. 2015).

In contrast, no child factors were related to use of *Reinforcement Approaches* at Bonferroni-adjusted thresholds. Therefore, in the present sample, other factors appear to drive variation in use of these parenting strategies. For *Reducing Uncertainty*, the largest contributor to variance was child *Intolerance of Uncertainty*. More modest contributions were detected from ASD severity and *Socially Inflexible Non*-*compliance,* dimensions which appear to be most linked to need for sameness and routine. Collectively, child factors explained up to 24% of the variance in this form of parenting.

Given the cross-sectional data, it is not possible to determine whether these observed relationships are causal. From a theoretical viewpoint, one might predict that child factors play a key role in driving both *Accommodation* and *Reducing Uncertainty*, since these strategies require parents to go out of their way to manage the environment around the child. Indeed, anecdotal reports from parents suggest that these strategies allow them to mitigate the effects of daily stress on their child with ASD. However, accommodation may also inadvertently reinforce problem behaviour. For example, giving in to the child (though it may be judged a necessity to avoid confrontation) might maintain or encourage a tendency for the child to escalate if things are not ‘on their terms’ (Lucyshyn et al. 2004). Constant accommodation may also reduce the child’s frustration tolerance, increasing the reinforcement value of reactivity as a means of maintaining control over their environment.

### Discipline-Oriented Parenting and Child Problem Behaviour

Since the PSQ did not include a measure specifically focused on discipline, we also explored the associations between child factors and *PBS Rules* and *PBS Discipline*. In the present sample, child factors were not significant predictors of either dimension collectively at Bonferroni adjusted thresholds (explaining < 5% variance), although ASD severity did appear to be a positive predictor of *Rules*. This discrepancy from previous work linking rules and discipline to child problem behaviour (Maljaars et al. 2014) may reflect the extremely high levels of reactivity in the present sample, even compared with other ASD samples (Mazefsky et al. 2018b), which could make discipline-oriented approaches difficult to implement.

A second possibility is that the parents who took part in this study construed their child’s problem behaviour differently from parents in other studies. If parents viewed difficult behaviour as related to the child’s incapacity to tolerate something, rather than wilful defiance, they may have been less likely to adopt discipline-oriented approaches. Indeed, we observed lower mean levels for discipline (by .5 of a standard deviation) and Rules (by 1.5 standard deviations) compared to Maljaars et al. (2014), which also included the Parenting Behaviour Scale. Cultural factors and differences in sample characteristics may play a role, but familiarity with the concept of extreme/‘pathological’ demand avoidance (‘PDA’) (a concept well known in the UK but not elsewhere) may also be relevant. In particular, descriptions of PDA include a formulation that places child anxiety as the driver of reactivity, and in doing so de-stigmatises the child and the parent for failures to enforce or conform with norms. Since interest in the concept of PDA largely centres on the UK, it is at present a culture-bound concept. Further cross-cultural comparisons in samples matched for symptom severity, taking into account residual cultural differences in terms of social pressure towards conformity, are required to investigate these possibilities further.

Finally, we also explored the relationships between *Inconsistent Discipline* and child problem behaviour-related variables. Here, modelled child factors predicted up to 8% of the sample variance, and *Demand*-*Specific Non*-*compliance* and *Extreme Demand Avoidance* significantly contributed to variance. However, given that the mean item score was 1.25, closest to ‘Almost never’, it could be argued that these associations reflect parents being either too overwhelmed to consistently maintain boundaries, or (viewed another way) more flexible, as opposed to a serious lack of consistency in the use of discipline in this sample.

## Caveats and Limitations

This study has a number of limitations. First, we did not have either a typically-developing or a non-ASD clinical control group, since the vast majority of those who did not meet inclusion criteria reportedly showed behaviours suggestive of ASD, but were not diagnosed. As such, we cannot draw conclusions about whether the strategies captured by the PSQ are more evident in parents of ASD children with problem behaviour than other populations, or whether our findings are specific to parents of ASD children.

Secondly, all measures were collected from a single respondent. Therefore, common rater-bias could have influenced the magnitudes of relationships that we detected between variables. Relying on informant report may be particularly problematic for variables such as child academic level and lack of independence in daily living activities. Therefore, future studies should explore the links between these factors and parenting strategies using multi-informant and clinical data.

Third, since most respondents were mothers, the present findings provide an incomplete picture of parenting dynamics in families of ASD children with problem behaviour. This is particularly relevant given reports of differences in parenting behaviours of mothers and fathers of children with ASD (Hirschler-Guttenberg et al. 2015). Future studies should attempt to collect data on parenting from both parents.

Fourth, we did not conduct clinical assessments using gold-standard diagnostic tools with our sample. Therefore, there may be some respondents with children who, had we assessed them, would not have met criteria for ASD, and also some who were not included in the analysis who would have met criteria. Misclassification of some children is unlikely to have led to spurious effects but may have added noise to our analyses. Furthermore, data on standardised assessments of IQ and verbal ability were unavailable. Therefore, further studies are needed in samples who have received a systematic in-person clinical assessment as part of the research protocol.

A further limitation is that parenting was measured using self-report, which may have been influenced by social desirability or inaccurate perceptions of one’s parenting behaviours. Future studies using alternative methods, such as in-home video recordings or observer ratings, are required to test the reliability of these findings.

In addition, the present sample may not be representative of parents of children with ASD in general in the UK. First, parents who took part were willing to complete lengthy questionnaires. Second, many of them had a particular interest in the concept of PDA. As such, our sample may overrepresent engaged and motivated parents who adopt more accommodative as opposed to authoritarian approaches. Therefore, these findings await replication in other parent samples recruited using different approaches. These considerations highlight the need to quantify parents’ cognitive models of the drivers of problem behaviour, beliefs about parenting, and parenting goals in studies of parenting behaviours in ASD.

Finally, in the present study, we analysed cross-sectional data. Therefore, it is not possible to be sure of whether and how child behaviour and parenting might influence each other. We speculate that child problem behaviour may drive accommodation, but accommodation may also maintain problem behaviour by positively reinforcing escalation in the child’s reactivity (e.g., Lucyshyn et al. 2004). Studies using lagged longitudinal designs and/or intervention methods are needed to explore the directionality of any causal effects.

## Practical Implications

Based on these and previous findings linking accommodation to anxiety and RRBs in ASD, one might hypothesise that encouraging parents to reduce accommodation might have benefits by increasing the opportunity for the child to habituate to stressful or aversive stimuli. Although this may be feasible for some, qualitative accounts suggest that for many, it may be necessary to first intervene to reduce child reactivity. This is because the need to prevent aggressive outbursts or intolerable levels of family stress appears to motivate and reinforce parents’ use of accommodation (Feldman et al. 2019; Lucyshyn et al. 2004). Furthermore, anxiety and escalation of behaviour found difficult or challenging if accommodation is not provided may have become a habitual child response in the context of certain family routines. Therefore, high levels of accommodation may be challenging to alter without intensive family support (e.g., Lucyshyn et al. 2007).

## Conclusion

The Parenting Strategies Questionnaire appears to capture three dimensions of parenting: *Accommodation*, *Reinforcement Approaches*, and *Reducing Uncertainty*, which were all commonly endorsed by parents of ASD children in our sample. Both *Accommodation* and *Reducing Uncertainty* subscales showed only modest associations with other parenting measures, suggesting that they complement and extend existing tools to assess parenting in ASD. Therefore, this measure might prove useful in intervention research to quantify reported use of these strategies.

To the best of our knowledge, ours is the first study to explore how parents may differentially adapt the strategies that they use to manage reactivity and problem behaviour depending on their ASD child’s behavioural profile. Child factors explained almost a third of the variance in *Accommodation*, and a quarter of the variance in *Reducing Uncertainty*. However, child factors were not related to variation in *Reinforcement Approaches*, raising the question of what factors drive individual differences in use of these strategies. Further work using longitudinal designs is needed to explore the direction of any causal links between parenting and child problem behaviour in families of children with ASD.

## Electronic supplementary material

Below is the link to the electronic supplementary material.

Supplementary material 1 (DOCX 27 kb)
