# Supplementary material for: Parenting Strategies Used by Parents of Children with ASD: Differential Links with Child Problem Behaviour
Source: J Autism Dev Disord. 2019 Nov 1;50(2):386–401. doi: 10.1007/s10803-019-04219-2 (PMC6994498; doi:10.1007/s10803-019-04219-2)
Supplement: Supplementary file 1 — Supplementary material 1 (DOCX 27 kb) [file 10803_2019_4219_MOESM1_ESM.docx]

**Supplementary materials: Parenting strategies used by parents of children with ASD: differential links with child problem behaviour**

**Supplementary Table 1:** Pearson correlation coefficients depicting relationships between discipline-related subscales child demographic, background and variables related to child problem behaviour.

**Supplementary Table 2:** Regression models predicting Discipline-related dimensions from child demographic factors and child problem behaviour-related.

**Supplementary Table 1**

|  | **Discipline-related subscales** | | |
| --- | --- | --- | --- |
|  | **Rules (PBS)** | **Discipline (PBS)** | **Inconsistent Discipline (APQ)** |
| **(A) Child demographic and background factors** | |  |  |
| Child age | .01 | .05 | -.09 |
| Child gender | -.08 | -.13 | .03 |
| Child lower academic level | .02 | .04 | -.03 |
| Child lack of independent living skills | -.02 | -.10 | -.03 |
| Child ASD severity (SCQ) | **.22**** | .05 | -.17** |
|  |  |  |  |
| **(B) Child problem behaviour-related variables** | |  |  |
| Reactivity (EDI) | .01 | -.01 | .17* |
| Demand-Specific (HSQ) | -.01 | .00 | **.22***** |
| Socially Inflexible (HSQ) | .01 | -.07 | .13 |
| Extreme Demand Avoidance (EDA) | -.06 | -.12 | **.24***** |
| Intolerance of Uncertainty (IU) | .01 | -.15* | .00 |

**Note: Pearson correlation coefficients depicting relationships between discipline-related subscales child demographic, background and variables related to child problem behaviour.** *p<.05, **p<.01, ***p<.001. Bonferroni adjustment across discipline-related subscales and child factors indicates an alpha threshold of p<.0017 (30 correlations calculated). Findings that survive Bonferroni correction are presented in bold. Parent age, SES, educational level, family size showed no significant associations with Discipline-related subscales, so no adjustments were made for these factors. For child-relevant variables that were not continuous, Spearman’s rank correlation coefficients are presented, and for continuous variables, Pearson’s correlation coefficients. Because some measures (*Extreme Demand Avoidance* and *Intolerance of Uncertainty*) showed negative skew, Spearman’s rank correlations were also calculated and compared with the Pearson’s estimates. Differences in correlation coefficients were $\leq$ .05.

**Supplementary Table 2**

|  | **Standardised regression coefficients for models containing each child problem behaviour variable** | | | | |
| --- | --- | --- | --- | --- | --- |
|  | **(1)** | **(2)** | **(3)** | **(4)** | **(5)** |
| **(A) Models predicting Rules** |  |  |  |  |  |
| Child age | -.01 | -.01 | -.01 | -.02 | -.01 |
| Child gender | -.13 | -.13 | -.12 | -.13 | -.13 |
| Child lower academic level | -.07 | -.07 | -.08 | -.08 | -.07 |
| Child lack of independent living skills | -.12 | -.12 | -.12 | -.11 | -.12 |
| Child ASD severity (SCQ) | **.28***** | **.28***** | **.28***** | **.28***** | **.28***** |
| (1) Reactivity (EDI) | .00 |  |  |  |  |
| (2) Demand-Specific (HSQ) |  | .01 |  |  |  |
| (3) Socially Inflexible (HSQ) |  |  | .01 |  |  |
| (4) Extreme Demand Avoidance (EDA) |  |  |  | -.04 |  |
| (5) Intolerance of Uncertainty (IU) |  |  |  |  | .00 |
| Variance in parenting explained | .04* | .04* | .04* | .05* | .04* |
|  |  |  |  |  |  |
| **(B) Models predicting Discipline** |  |  |  |  |  |
| Child age | .06 | .06 | .06 | .05 | .08 |
| Child gender | -.25 | -.25 | -.23 | -.22 | -.19 |
| Child lower academic level | .06 | .06 | .05 | .05 | .02 |
| Child lack of independent living skills | -.17* | -.20* | -.15 | -.15* | -.14 |
| Child ASD severity (SCQ) | .07 | .08 | .08 | .07 | .10 |
| (1) Reactivity (EDI) | .03 |  |  |  |  |
| (2) Demand-Specific (HSQ) |  | .07 |  |  |  |
| (3) Socially Inflexible (HSQ) |  |  | -.03 |  |  |
| (4) Extreme Demand Avoidance (EDA) |  |  |  | -.08 |  |
| (5) Intolerance of Uncertainty (IU) |  |  |  |  | -.14* |
| Variance in parenting explained | .01 (n/s) | .02 (n/s) | .01 (n/s) | .02 (n/s) | .03 (n/s) |
|  |  |  |  |  |  |
| (**C) Models predicting Inconsistent Discipline** | |  |  |  |  |
| Child age | -.07 | -.09 | -.09 | -.07 | -.11 |
| Child gender | .00 | .01 | .04 | .00 | .03 |
| Child lower academic level | .02 | .03 | .02 | .05 | .03 |
| Child lack of independent living skills | .00 | -.09 | -.02 | -.01 | .02 |
| Child ASD severity (SCQ) | -.20** | -.18* | -.19* | -.17* | -.19* |
| (1) Reactivity (EDI) | .18** |  |  |  |  |
| (2) Demand-Specific (HSQ) |  | **.27***** |  |  |  |
| (3) Socially Inflexible (HSQ) |  |  | .15* |  |  |
| (4) Extreme Demand Avoidance (EDA) |  |  |  | **.22***** |  |
| (5) Intolerance of Uncertainty (IU) |  |  |  |  | .06 |
| Variance in parenting explained | .05* | **.08***** | .04* | **.06**** | .02 (n/s) |

**Note: Regression models predicting Discipline-related dimensions from child demographic factors and child problem behaviour**. Separate regression models were run for each child problem behaviour-related dimension and each parenting approach on standardized variables (gender not standardised). For each parenting strategy, Model (1) contained Reactivity, (2) Demand-Specific, (3) Socially Inflexible, (4) Extreme Demand Avoidance, (5) Intolerance of Uncertainty. *p<.05, **p<.01, ***p<.001. Bonferroni adjustment for the overall significance of the regression models indicates an alpha threshold of p<.003 (15 models estimated). Bonferroni correction within models containing six independent predictors indicates an alpha threshold of p<.008. Findings that survive Bonferroni correction are presented in bold.
